# Advancing health policy and systems research and analysis: new frontiers, renewed relevance

**DOI:** 10.1093/heapol/czag014

**Published:** 2026-02-17

**Authors:** Aku Kwamie, Lucy Gilson, Rachidatou Compaore, Keith Cloete, Fadi El-Jardali, Idil Shekh Mohamed, Sassy Molyneux, Sudha Ramani, Helen Schneider, Prashanth N Srinivas, Goran Tomson, Benjamin Tsofa, Kumanan Rasanathan

**Affiliations:** Alliance for Health Policy and Systems Research, World Health Organization, Geneva, Switzerland; Health Policy and Systems Division, University of Cape Town, Cape Town, South Africa; Institut Africain de Sante Publique, Ouagadougou, Burkina Faso; Department of Health and Wellness, Western Cape Province, Cape Town, South Africa; Knowledge to Policy Centre, American University of Beirut, Beirut, Lebanon; Alliance for Health Policy and Systems Research, World Health Organization, Geneva, Switzerland; Nuffield Department of Medicine, Oxford University, Oxford, United Kingdom; Independent consultant, Delhi, India; School of Public Health, University of the Western Cape, Cape Town, South Africa; Center for Health Systems, Institute of Public Health, Bengaluru, India; Department of Global Public Health, Karolinska Institutet, Stockholm, Sweden; KEMRI-Wellcome Trust Research Programme, Kilifi, Kenya; Alliance for Health Policy and Systems Research, World Health Organization, Geneva, Switzerland

**Keywords:** health policy and systems research, health systems, analysis, knowledge, practice, capabilities, theory, systems thinking

## Abstract

Health systems are at a crossroads. Globally, health systems are straining under the weight of responding to persistent and emergent challenges. Uncertainties in health financing, service delivery, new technologies and disease burdens are hindering health systems abilities to maximize collective and scaled action to achieve health equity and social justice. In March 2025, a group of health policy and systems experts were convened by an organization, to consider the ‘new frontiers’ of the field in the context of shifting global and national landscapes. Deliberations centred on the critique that health policy and systems research (HPSR) need to restate its core foundations, better articulate its impacts in real health systems and policy processes, while defining its role within or apart from ‘global health’. Six frontiers were identified: new institutional forms of HPSR beyond academic settings; more fully theorized and hypothesized studies that go beyond descriptive; more applies systems thinking; new educational models to support analysis, networking and systems leadership; more domestic financing for HPSR; and genuine engagement with a new set of health system development actors. For HPSR to remain relevant, strengthening the science and practice of how diverse actors engage to bring about collective action for health equity and social justice is imperative. The current global geopolitical, financing, and planetary shifts, while critical, present an opportunity for these new frontiers in HPSR to deepen the impact of the field.

Key messages

- The concerns of health policy and systems research with health equity and social justice make the field, in the current context of reduced development assistance for health, even more relevant than ever.
- A role of health policy and systems research in this new global context persists, though its boundaries need to be more sharply defined.
- These ‘new frontiers’ for health policy and systems research include several shifts, namely: more practice and experientially-rooted knowledge and analysis; better theorized and hypothesized methodological approaches; explicit use of systems science and complex adaptive properties; development of new education programmes that focus on training for leadership and facilitation skills, boundary-spanning, and enabling systemic change; increased domestic financing for health policy and systems research; and intentional engagement with an expanded cast of actors across public and private spheres.

## Health policy and systems research: more important than ever

Health policy and systems research (HPSR), as an applied field, is more relevant than ever. HPSR seeks to understand and improve how societies organize themselves in achieving collective health goals, and how different actors interact in the policy and implementation processes to contribute to policy outcomes (Alliance for Health Policy and Systems Research). At its core, HPSR is centred on health equity and social justice. Moreover, health systems enable collective and scaled action to support change towards the goals of achieving health equity and social justice. Yet, health systems face systemic constraints, as well as both persistent and newly emerging threats that, taken together, limit health systems’ abilities to respond collectively. The COVID-19 pandemic demonstrated this and challenged researchers to respond differently in how they generate their research (Gilson et al. 2020). HPSR seeks to support health systems to develop the capabilities of renewal and reinvention in response not only to challenges, but also to opportunities that emerge from all health system levels. This ‘why’ of HPSR, is brought into even sharper relief in the current global context where the pressures on health systems exacerbate existing health inequities for service users and providers alike (Rasanathan 2024). Dramatic reductions in development assistance for health allied to stagnating or reducing domestic fiscal space for health, has created immediate ‘financing cliffs’ for the resourcing of health systems in many low- and middle-income countries (LMICs) (Rasanathan et al. 2025). However, such drastic change also opens up structural opportunities for rethinking programming and service delivery, and widening policy decision-spaces (Sheikh and Schneider 2025). Table 1 summarizes the field’s achievements and enduring challenges.

**Table 1:** Dimensions of HPSR’s achievements and enduring challenges.

Field dimension	Achievement	Enduring challenges
Values	HPSR explicitly endorses equity and social justice as foundational values that must guide research.	Tendency towards prioritizing technical interventions over values-driven approaches. There is need for the field to better articulate (1) the domains of equity research, shifting current discourse away from diagnosis and monitoring, towards research that can catalyse policy and systems change; (2) the process of research that can redress equity; and (3) expanding the methods used in equity research.
Development and use of theory	HPSR recognizes health systems as complex systems that are embedded in wider socio-political-economic systems.	Research in the field has not always engaged deeply with systems theories (such as systems science/complexity theory, or policy analysis theory), and can contribute more to theory generation to strengthen its insights and relevance.
Research	HPSR has advanced research that is relevant to policy and practice, and has encouraged contextually-grounded studies in LMICs.	Knowledge hierarchies, conventional forms of academic research, and positivist methodological approaches still dominate the field.
Capacity	HPSR is now recognizably practiced in many more LMICs than 20 years ago, with more institutions offering training programmes.	Sustaining a critical mass of HPSR practitioners in country, attaining leadership positions, and advancing competencies beyond research, and new organizational capabilities.
Policy and practice engagement	HPSR has always emphasized the importance of engagements with policy makers and practitioners.	There has been less funding for deeper, meaningful and long-term engagements. Further, in today’s emerging policy landscape, there is growing influence of new private actors (such as the digital and tech industry) as well as increasingly complex forms of governance required to address the social determinants of health. This presents new arenas for engagement for HPSR scholars and practitioners.
New topics	HPSR is well suited to grapple with the emerging issues are facing health systems, health policies, and research, including the nexus between climate and health; changing epidemiology of diseases in LMICs; impacts of wars and conflicts on already fragile health systems; and the rise of AI and the digital transformation.	Longstanding themes—such as governance and leadership—remain relevant, and newer health and health system challenges should not displace these, but rather widen the HPSR perspective for adaptive capacities.
Cuts to foreign aid and science funding	Funding for the field of HPSR has grown. Further, there is increased focus on equitable partnerships with LMICs, and researchers seek to redress structural imbalances.	Global funding cuts present very real challenges not only to health system functioning but to further growth in the field. A large proportion of funding and major research initiatives are still concentrated in the universities of high-income countries.

Abbreviations: AI: artificial intelligence; HPSR: health policy and systems research; LMIC: low- and middle-income country.

In the midst of these global challenges, stronger articulation of HPSR offers opportunities to act. Understanding health systems as complex systems, embedded in wider systems further underscores their nature as complex adaptive systems (CAS), in the sense of people and teams connected by processes, behaviours, and relationships. It is important to recognize that health systems are driven by their goal of collective action for health and well-being, which as the catalyst for achieving better outcomes across multiple organizations and actors. The essence of HPSR’s relevance can be captured in the following three points:

1. *Whole-system changes are needed within health systems, from healthcare systems to social determinants of health*. HPSR supports capacities and capabilities required to act across the entirety of the health system as a CAS—a coherent whole with intersecting components and deeper elements of mindsets, values and norms that influence system properties such as self-organization and emergence—and not just interactions within or between discrete ‘building blocks’. This refers to both ‘system-wide’—broad, national-level approaches to service delivery across sectors, in which health is embedded—and ‘system-deep’—fundamental interventions based on how decisions get made in practice, through shared goals, relational governance and distributed leadership, that address structural inequalities.
2. *Systems science is needed to respond to the complexity inherent to many health system challenges*—what Rittel and Webber refer to as ‘wicked problems’ (Rittel and Webber 1973)—problems that are defined as context-specific, hard to formulate, don’t have true or false solutions, and have multiple and interdependent causes.
3. *Policy science is needed to recognize the political nature and power dynamics of systems change*. Governance—the ability to bring about collective action—remains an enduring concern, and an under-studied topic for HPSR (Alliance for Health Policy and Systems Research 2008, Bigdeli et al. 2020).

## HPSR’s new frontiers?

Given the current global geopolitical, technological and planetary health shifts, what is the role of HPSR? What is its future? What are the borders of HPSR’s new frontiers? In March 2025, a group of HPSR researchers and policymakers from across Africa, Asia, Eastern Mediterranean and Europe was convened by the Alliance for Health Policy and Systems Research to deliberate on these questions, with a view to reflecting on HPSR’s core foundations, and recommending ways forward to sustain a critical mass of HPSR actors, develop in-country competencies and capabilities for HPSR, and better identify and articulate the impacts of HPSR, particularly in the current context. Below, we put forth six considerations that have the potential to sharpen the science, practice, and development of HPSR, enlarge its stakeholder base, and improve its stability, recognition and impact.

1. *New institutional forms of HPSR not limited to academic settings*

There is an urgent need to combine formal research, and knowledge derived from practice. HPSR’s traditional locus within academic settings (i.e. universities and research centres) has perhaps assumed that the best solutions to health systems development and transformation are found through formal academic research, or, that such research is primarily an act of formalized knowledge collection by researchers, rather than an interplay of formal research with the existing and tacit knowledges existent within and across health system actors, including practitioners, policymakers and community members. Beyond university-based research, there is a need to include research approaches that are less traditionally academic, both in terms of methods and elitism, and more rooted in practice and experiential knowledge. Relieving HPSR from the weight of perceptions of research as being slow and elite could promote better use of routine data and practice within organizations, and encourage learning health systems (Witter et al. 2022).

Relatedly, valorizing and strengthening individual and organizational capacities for health policy and systems *analysis*—real-time use of data and evidence for learning and improvement that might not be termed ‘research’ from an academic perspective—can enable health systems synergies that bridge formal research and practice knowledge. Indeed, it may be more useful to think in terms of ‘analysis’ rather than only of research, to embrace practitioners and other non-academics in the HPSR field. Efforts in embedded implementation research have sought to achieve this, but more work is needed. Further, forms of research that can be termed praxis, inquiry or local analysis—including action learning approaches—can come under the research umbrella, and better encompass the routine data and behaviours that make up the decision-making practice of health managers, planners, programme analysts and leaders within health systems. Within academia, this could mean widening the field of public health (where HPSR is most commonly located) to embrace public health science that more greatly appreciates application of organizational, systemic, political and policy understandings as part of research and real-time analysis. Digital transformation and the increase of artificial intelligence may offer solutions to facilitate these shifts in health systems and move HPSR beyond academia.

2. *More systematic, less descriptive studies, that are more fully theorized and operationalizable*

Methodological discomforts persist within HPSR. First, much of the research emanating from the field is still descriptive. Such studies diminish the scope and potential of HPSR by preventing advancements of the field towards deeper insights into how to support health systems change. Better theory can achieve greater explanatory focus, and move HPSR away from simple description towards deeper analysis of how systems change can be supported and achieved. More theory-building, hypothesizing, and assumption-testing is essential. Second, there is need to further develop practice-informed theory, hypotheses and approaches based on local, contextually-relevant science and real-time analysis. Importantly, practice-based theory should be applied in intervention design, implementation and evaluation. In the case of research that can advance equity, expanded practice-based methods that privilege lived experience and research processes that themselves redress equity can help to shift current equity discourse from diagnosis and monitoring of equity in societies, towards research that can catalyse policy and systems change.

3. *More applied systems thinking to better inform systems praxis*

Despite widespread assent of the importance of systems thinking in HPSR, systems thinking *application* has been rather superficial. The HPSR literature remains dominated by calls for more systems thinking, while lacking in corresponding empirical examples (Kwamie et al. 2021). This is partly explained by HPSR’s over-reliance on systems ‘lenses’, rather than explicitly drawing from complexity theories and systems science methodologies—including participatory methods that go beyond modelling—that can make sense of the complexity that arises not from the technical aspects of health system interventions, but from divergent stakeholder worldviews. Deeper understanding of system typologies and CAS properties (i.e. emergence, self-organization, delays, feedback, path dependence) will improve HPSR design and evaluation, as well as appreciation for which systems thinking methods are suitable, when. Vitally, better considerations of time and systemic risk as CAS properties will have implications for developing understanding of longitudinal causal pathways across health system levels—much-needed in responding to the interconnected and cascaded effects of multiple global challenges (polycrisis)—and a current shortcoming in the HPSR field. This is particularly critical in the current context of multiple, overlapping global challenges, where better application of systems thinking, systems science methods and complexity theory can elucidate underlying patterns and identify leverage points to inform policy actions (Kwamie et al. 2024).

4. *New educational interventions to support analysis, networking, advocacy and systems development*

To nurture future generations of health policy and systems analysts and researchers who will sustain and develop the field in the ways proposed herein, it will be important to strengthen education for HPSR. This involves considering both training modalities (how) and competencies (what) that need strengthening. For instance, there remain too few dedicated post-graduate health system training programmes, especially in LMICs. New education programmes that begin even at the undergraduate level must offer analytic skills’ development, applied systems thinking, advocacy, and policy analysis, in research, analysis, and practice (Erasmus et al. 2016). Such educational programmes must also move outside of the classroom, to offer workplace-based learning. Relatedly, new forms of leadership development that (1) focus on applied systems thinking, reflective practice, and facilitation skills; (2) develop skills, aptitudes and mindsets needed to work across disciplinary and organizational boundaries; and (3) can enable whole-systems change, are needed. Systems leadership—the ability to understand the larger system and drive collective action to catalyse systems-level change—needs fostering, not only in training programmes, but also in programme implementation and reshaping organizational structures (Senge et al. 2015, Dreier et al. 2019, Gilson et al. 2023). Additionally, skills in advocacy can help researchers and practitioners with strategic communications, and foster engagement, dialogues and trust.

5. *Stronger domestic financing of HPSR to support national and regional networks*

HPSR remains chronically underfunded through domestic sources, compared to clinical and biomedical research across all global regions (Lamba et al. 2021). In most LMICs, HPSR is highly dependent on aid—which in the current global health financing crisis with aid withdrawals means that hard-won progress in HPSR capacity is fragile and under threat. Domestic financing for HPSR is therefore an urgent priority. The challenges of securing more domestic financing for HPSR include poor understanding of HPSR and of its potential impact; the variability in whether countries have dedicated or disaggregated budget lines for health research and HPSR; consensus definitions of HPSR that delineate which activities are (or are not) covered; recognition of HPSR as a field; and information systems that consistently collect data on HPSR-related indicators (Alliance for Health Policy and Systems Research 2021). In all countries, HPSR practitioners in policy, academic and other spheres must advocate for a larger share of health and social research funding, even as all research funding will be under pressure as countries struggle to finance basic health services and commodities. Advocacy could also be oriented towards urging country governments to earmark funding for the evaluation of policies and intervention as a source of HPSR funding.

6. *Engagement of the HPSR community with the shifting meaning of health systems development and transformation*

If HPSR is to stay relevant, then the scope of actors and organizations the HPSR community will have to engage with must expand. At national and global levels, this includes researchers from other disciplines; organizations beyond academia and government; private sector actors beyond health; and community actors beyond those who have been co-producers and beneficiaries of HPSR inquiry. Government science advisors, health policy diplomats, tech entrepreneurs, urban planners, and ecologists are but a few of the differentiated actors to be considered. In addition to ‘who’, ‘how’ HPSR engages with new audiences and partners is of great importance. Inherent in this expanded engagement will be a necessary demonstration, rather than simple assertions, of the relevance of HPSR for health systems in need of transformation. Greater use of frameworks rooted in solidarity can offer a means of addressing global vulnerabilities and local responsiveness aggravated by demographic, environmental, technological and geopolitical shifts (Tomson et al. 2021).

## Conclusions

In the current global moment, HPSR has a role to play in creating spaces within country structures to bring together differing perspectives that can contribute to the collective action for health equity and social justice. HPSR provides solutions to the challenges of health systems development and transformation as a field of practice that can facilitate the exchange of learning and experience across boundaries—within national settings and across national settings—to spread learning and support the adaptation of innovation and experience across settings. This will necessarily involve strengthening the science and practice of how diverse health actors interact to bring about collective action, and analysing how such action can be cascaded upwards from individual to organizational to system levels. HPSR as a field of practice can support a widened understanding of research and real-time analysis by bringing systematic and rigorous research processes into productive relationship with the tacit and experiential knowledge of practitioners about how systems work, to generate actionable institutional change.

The current global shifts, while critical, also present opportunity for new spaces and approaches to emerge. Developing HPSR and real-time analysis capabilities at country-level has the potential for shaping new organizational forms of HPSR practice. The new frontiers of HPSR, then, are about how to balance these multiple tensions: advancing theory-building *and* testing; ensuring theoretical rigour *and* policy responsiveness; knowledge generation as a formalized process *and* a process of co-construction informed through practice; education as supporting skills and systems development. This can bring about a future for HPSR that is inclusive and impactful.

## Data Availability

There are no new data associated with this article.

## Reflexivity statement

The authors include seven women and six men. The group spans multiple levels of seniority, including one early-career, six mid-level and six senior career stage. There are nine academics, one policymaker, and three global organization staff in the group. All authors are health policy and systems experts, with extensive experience leading health policy and systems research at subnational, national and global levels. Eight of the authors are embedded researchers living and working in low- and middle-income settings.
